# An integrative and citizen science based approach to the rediscovery and redescription of the only known high-altitude endemic Pill Millipede, *Glomeris aurita* Koch (Diplopoda, Glomerida)

**DOI:** 10.7717/peerj.5569

**Published:** 2018-09-13

**Authors:** Thomas Wesener

**Affiliations:** Center for Taxonomy and Evolutionary Research (Section Myriapoda), Zoological Research Museum A. Koenig, Leibniz Institute for Animal Biodiversity, Bonn, Germany

**Keywords:** Integrative taxonomy, Rediscovery, Citizen science, Bergamasque alps, High-altitude endemics, Color morphs, Biodiversity, CT scan

## Abstract

The pill millipede species *Glomeris aurita* Koch, 1847 remained of relative unknown origin and appearance until its recent rediscovery in samples from the Bergamasque Alps, northern Italy. In order to provide an integrative redescription and accurate identification of the high-altitude microendemic *G. aurita*, COI barcode sequences from three individuals coming from two different localities were obtained. These sequences are compared with those of the syntopic endemic *G. oblongoguttata* Verhoeff, 1894, the widespread black morph of *G. romana* Verhoeff, 1900, as well as several widespread species including *G. marginata* Villers, 1789, *G. connexa* Koch, 1847, and *G. klugii* Brandt, 1833, which have rare colour morphs that exhibit some similarity to *G. aurita*. To rule-out any identity confusion of *G. aurita* with other high-altitude or little-known Italian *Glomeris*, specimens of *G. transalpina* Koch, 1836, *G. oropensis* Verhoeff, 1934, and *G. primordialis* Verhoeff, 1932 were also added to the dataset. Altogether, 24 sequences were compared. Morphologically, the specimens of *G. aurita* were studied utilizing scanning electron microscopy as well as non-invasive micro-CT technology. The distribution of both Bergamasque endemics, *G. aurita* and *G. oblongoguttata*, could be mapped and compared utilizing samples from the Museo civico di Scienze Naturali di Bergamo, as well as photographic evidence from an Italian naturalist forum. *G. aurita* has a very short active period and is the first known pill millipede species restricted to mountain tops and cold places, possibly representing a Nunatak survivor.

## Introduction

More than 160 years ago Koch described the endemic species *Glomeris aurita* (1847) from ‘upper Italy’. While this species was mentioned in the literature by Brölemann (1895), and listed as a species occurring in caves east of Lago di Como by Manfredi (1932), definite specimen-based records have been absent since 1895. Verhoeff, Attems and Strasser, probably the most prolific researchers and collectors of the northern Italian millipede fauna, list this species (Verhoeff, 1936; Attems, 1949), but mention that they had never seen any specimens. In the most recent atlas, *G. aurita* is listed as a potential cavernicole (Kime & Enghoff, 2011). With the types apparently lost (see below), no identified specimen of *G. aurita* was readily present in any natural history collection. Indeed, the colour pattern figured in the first description of *G. aurita*, as well as the striae on its thoracic shield, both key characters for the identification of *Glomeris* species (Hoess, 2000), are very similar to the black colour morph of one of Italy‘s most widespread pill millipede species (Kime & Enghoff, 2011), *Glomeris romana* (Verhoeff, 1900). Such dark colour mophs are common in poikilothermic animals (Clusella-Trullas, Van Wyk & Spotila, 2009). The question whether *G. aurita* represents a true Italian endemic or a high altitude black colour morph of the widespread *G. romana* seemed impossible to answer.

*G. aurita* Koch, 1847, is the oldest of the endemic Italian pill millipedes (order Glomerida) of the genus *Glomeris* Leach, 1814. Italy is particularly rich in Glomerida endemics, with 21 country endemics known (Strasser & Minelli, 1984; Kime & Enghoff, 2011), more than in all other European countries combined. Most of these endemics (14) are restricted to northern Italy. *G. aurita* Koch, 1847, along with seven species of the *G. dorsosanguine* species-group (*G. dorsosanguine* Verhoeff, 1906, *G. judicaria* Verhoeff, 1936, *G. longaronensis* Verhoeff, 1930, *G. sanguinicolor* Verhoeff, 1909, *G. schubarti* Verhoeff, 1931, *G. solis* Verhoeff, 1934, and *G. strasseri* Verhoeff, 1929), is one of eight *Glomeris* species microendemic to north-eastern Italy that remains of dubious status, most of them known only from their first description. Recently, progress has been made in the redescription of other forgotten pill millipede species. One of these ‘forgotten’ species, *G. apuana* Verhoeff, 1911 was revised with the help of barcode data (Wesener, 2015b). Additionally, a recent integrative revision investigated four endemics, *G. oropensis* Verhoeff, 1934, *G. primordialis* Verhoeff, 1932, *G. oblongoguttata* Verhoeff, 1894 and *G. larii* Verhoeff, 1921 belonging to the *Glomeris klugii* species-group, and discovered *G. larii* as a synonym (Wesener & Conrad, 2016).

An opportunity to use a molecular analysis to clarify whether *G. aurita* represents a true Italian endemic unexpectedly arose after Axel Schönhofer (Schönhofer et al., 2015), a renowned Opiliones expert, shared specimens fitting the original description (Koch, 1847) of *G. aurita*, and the drawings published in Koch’s atlas (Koch, 1863). The specimens were collected as by catch from alpine meadows (>1,500 m) in the Bergamasque Alps in northern Italy. Unfortunately, the samples were too old by the time the molecular analysis was planned and a DNA extraction was unsuccessful.

The possibility of clarifying the status of *G. aurita* motivated a July 2014 expedition to the three localities of the rediscovered specimens. However, the exact collection spots were the last places still covered by snow fields and no specimens were found. The author then enlisted the help of Marco Valle and his colleagues from the Bergamo Museum to obtain relatively fresh specimens suitable for a molecular analysis of *G. aurita*. These specimens could be compared with sequences obtained from the black colour morph of *G. romana*, as well as several other widespread species with black colour morphs such as *G. connexa*, the black *G. marginata*, and *G. klugii*, including another endemic of the Bergamasque area (Hoess & Scholl, 1999), *G. oblongoguttata*, as well as other high-altitude *Glomeris* species, such as *G. transalpina* (Koch, 1836), *G. oropensis* (Verhoeff, 1932), and *G. primordialis* (Verhoeff, 1936). Citizen science plays an even greater role in the digital age through large websites (iNaturalist, 2018) visited by thousands of people every day, many providing image vouchers for numerous species. However, most of the recorded species are those that are large enough (usually >10 mm), can easily be seen, and can be identified based on photographs, something impossible for the majority of soil arthropods such as millipedes. Luckily, for pill millipedes of the genus *Glomeris*, the color pattern is an important character to distinguish species. Regarding *G. aurita*, citizen scientists, including Fernando Scarlassara, from the Italian naturalist online forum ‘NaturaMediterraneo’ (NaturaMediterraneo, 2018) have provided further locality data of the species that greatly expands its known distribution. As these citizen scientists did not collect and conserve encountered specimens, photographic evidence can be used as vouchers for the localities.

This study shows how molecular barcoding techniques can correctly distinguish between colour morphs and separate species of Italian pill millipede species. The importance of citizen science, the passing on of by catch, and the value of the examination of the collections of local museums allows the outline of the distribution and the ecological niche of hard-to-collect species such as high-montane endemics.

## Material and Methods

### Morphological analysis

Aside from the coloration pattern, the number of thoracic shield striae are the most important taxonomic character in the genus *Glomeris*. In order to assess the intraspecific variation of the striae, they were counted for 58 specimens from 10 localities of *G. aurita* as well as for 59 specimens from 21 localities of the syntopic *G. oblongoguttata*. Specimens of both sexes as well as juveniles were included (Supplementary information 1).

For scanning electron microscopy (SEM), samples were transferred to 100% ethanol, dried overnight, sputtered with gold and observed under a Hitachi S4260 scanning electron microscope. As the taxonomically relevant characters for species of the genus *Glomeris* have not been explored in detail, as many potentially important characters as possible are illustrated.

To assess the utility of less invasive techniques to study the taxonomically relevant characters of pill millipedes, a micro CT scan of a specimen of *G. aurita* was also conducted. Obtained results were compared with those of the SEM. For micro-CT, the specimen was scanned in ethanol inside the collection vial in a Bruker Skyscan 1,272 system. Settings were: Source Voltage =60 kV, Source Current = 166 µA, Exposure = 915 ms, Rotation of 360° in Rotation Step of 0.2°, Frame Averaging = 7, Random Movement = 15, Flat Field Correction ON, Geometrical Correction ON, Filter = Al 0.25 mm. Reconstruction and thermal drift correction was performed in NRecon 1.7.0.4 (Bruker microCT). The resulting image stack was cropped in ImageJ (Abràmoff, Magalhães & Ram, 2004), preserving the original pixel sizes. Subsequent volume rendering and measurements were done in Drishti 2.6 (Limaye, 2012).

### Biogeographic data sampling via citizen scientists and museum collections

All Glomerida material of the vast collections (>200 vials) of the local Italian museum, the Museo di Scienze Naturale “E. Caffi”, in Bergamo was obtained on loan and determined to species-level (see below).

A further five localities for *G. aurita* were discovered via posts in an Italian naturalist forum (Natura Mediterraneo) and thankfully mapped by F. Scarlassara. For these five localities (detailed below) only photographic evidence for the occurrence of the species exists.

### DNA extraction, amplification and sequencing

Generally, the DNA protocol was similar to that employed in earlier studies (Wesener, 2015a; Wesener, 2015b), utilizing the degenerated HCO-JJ/LCO-JJ primer pair (Astrin & Stüben, 2008). The three new sequenced have been uploaded to Genbank under the code MH574905 –MH574907 (Table 1).

**Table 1:** Analyzed species, voucher numbers, locality and Genbank information.

**Species**	**Voucher #**	**Locality**	**Genbank #**
*Glomeris marginata*	ZFMK MYR009	Germany, Bonn-Kessenich, Venusberg	FJ409909
*Glomeris connexa* 1	ZSM MYR00372	Italy, Lombardia, Sondrio.	JN271879
*Glomeris connexa* 2	ZSM MYR00027	Germany, Bavaria, Andechs.	HM888096
*Glomeris connexa* 3	ZSM MYR00028	Germany, Bavaria, Scheidegg.	HM888097
*Glomeris connexa* 4	ZSM MYR00026	Germany, Bavaria, Garmisch.	HM888095
*Glomeris transalpina* 1	ZFMK MYR2609	Switzerland, Wallis, Riederalm, E. Riederfurka.	KX714038
*Glomeris transalpina* 2	ZFMK MYR2636	Switzerland, Wallis, Simplonpass.	KX714039
*Glomeris oropensis*	ZFMK MYR4534	Italy, Piemonte, Biella, NW of Oropa.	KX714040
*G. romana marinensis*	ZFMK MYR797	San Marino, San Marino Stadt.	KX714036
*G. romana romana*	ZFMK MYR1471	Italy, Umbria, Perugia, NE Gosparini,.	KX714037
*Glomeris oblongoguttata* 1	ZFMK MYR4567	Italy, Lago di Iseo, Pisogne.	KX714041
*Glomeris oblongoguttata* 2	ZFMK MYR4570	Italy, Lago di Iseo, Pisogne.	KX714042
*Glomeris oblongoguttata* 3	ZFMK MYR4564	Italy, Lago di Iseo, Pisogne.	KX714043
*Glomeris primordialis* 1	ZFMK MYR4744	Italy, Piemonte, Biella, Pollone - Favaro.	KX714046
*Glomeris primordialis* 2	ZFMK MYR4741	Italy, Piemonte, Biella, Pollone - Favaro.	KX714047
*Glomeris klugii* 1	ZFMK MYR4522	Germany, NRW, Hagen-Holthausen.	KX714057
*Glomeris klugii* 2	ZFMK MYR4520	Germany, NRW, Hagen-Holthausen.	KX714058
*Glomeris klugii* 3	ZFMK MYR4768	Germany, Siebengebirge, Löwenburg.	KX714060
*Glomeris klugii* 4	ZFMK MYR4769	Germany, Siebengebirge, Löwenburg.	KX714061
*Glomeris klugii* 5	ZFMK MYR4729	Italy, Riva, Valley of W. Riva.	KX714065
*Glomeris klugii* 6	ZFMK MYR4728	Italy, Riva, Valley of W. Riva.	KX714066
*Glomeris aurita* 1^*^	ZFMK MYR 2867	Oltre il Colle, Valle d’Arera, 2,000 m	MH574905
*Glomeris aurita* 2^*^	ZFMK MYR 2866	Oltre il Colle, Valle d’Arera, 2,000 m	MH574906
*Glomeris aurita* 3^*^	ZFMK MYR 2641	Oltre il Cole, Mt. Alben, Piano Palla, locality Forca Larga, 1,480 m.	MH574907

**Notes.**

* marks newly sequenced specimens.

### Alignment and distance analysis

Sequences were aligned by hand in Bioedit (Hall, 1999). The final dataset included 24 nucleotide sequences with 657 positions (three newly sequenced and 21 from Genbank, see Table 1). The alignment is provided as (Supplementary information 2). Phylogenetic analyses were conducted in MEGA6 (Tamura et al., 2013). A Modeltest, as implemented in MEGA6 (Tamura et al., 2013), was performed to find the best fitting maximum likelihood substitution model. Models with the lowest BIC scores (Bayesian Information Criterion) are considered to describe the best substitution pattern. Codon positions included were 1st + 2nd + 3rd. Modeltest selected the Hasegawa–Kishino–Yano model (Hasegawa, Kishino & Yano, 1985) with gamma distribution and invariant sites as best fitting model (lnL −2,692.89, Gamma 0.11, R 5.29, Freq A: 0.265, T: 0.39, C: 0.141, G: 0.204).

The evolutionary history was inferred by using the maximum likelihood method based on the selected Hasegawa–Kishino–Yano model (Hasegawa, Kishino & Yano, 1985). The tree with the highest log likelihood (−2690.02) is shown. Initial tree(s) for the heuristic search were obtained automatically by applying Neighbor-Join and BioNJ algorithms to a matrix of pairwise distances estimated using the Maximum Composite Likelihood (MCL) approach, and then selecting the topology with superior log likelihood value. A discrete Gamma distribution was used to model evolutionary rate differences among sites (five categories (+G, parameter = 0.1129)). The tree is drawn to scale, with branch lengths measured in the number of substitutions per site. The analysis involved 24 nucleotide sequences. Codon positions included were 1st + 2nd + 3rd. The bootstrap consensus tree inferred from 1000 replicates (Felsenstein, 1985) is taken to represent the evolutionary history of the analyzed taxa. The tree is drawn to scale, with branch lengths measured in the number of substitutions per site.

The number of pairwise base differences per site were calculated in MEGA6 (Tamura et al., 2013). Codon positions included were 1st + 2nd + 3rd. In the distance analysis, all positions containing ‘N’s were removed for each sequenced pair. There were a total of 657 positions in the final dataset. The results are shown in Table 2.

**Table 2:** The number of base differences per site from between sequences are shown. The analysis involved 24 nucleotide sequences. Codon positions included were 1st+2nd+3rd. All ambiguous positions were removed for each sequence pair. There were a total of 657 positions in the final dataset. Intraspecific distances in bold. Asterisks mark specimens sequenced for this study.

**#**	**Species**	**1**	**2**	**3**	**4**	**5**	**6**	**7**	**8**	**9**	**10**	**11**	**12**	**13**	**14**	**15**	**16**	**17**	**18**	**19**	**20**	**21**	**22**	**23**
1	*Glomeris marginata*																							
2	*Glomeris connexa 1*	0,139																						
3	*Glomeris connexa 2*	0,143	**0,017**																					
4	*Glomeris connexa 3*	0,143	**0,017**	**0,000**																				
5	*Glomeris connexa 4*	0,143	**0,017**	**0,000**	**0,000**																			
6	*Glomeris transalpina 1*	0,146	0,134	0,137	0,137	0,137																		
7	*Glomeris transalpina 2*	0,146	0,134	0,137	0,137	0,137	**0,000**																	
8	*Glomeris oropensis*	0,159	0,151	0,151	0,151	0,151	0,099	0,099																
9	*G. romana marinensis*	0,146	0,145	0,151	0,151	0,151	0,151	0,151	0,154															
10	*G. romana romana*	0,146	0,145	0,151	0,151	0,151	0,151	0,151	0,154	**0,000**														
11	*Glomeris oblongo 1*	0,152	0,148	0,146	0,146	0,146	0,110	0,110	0,114	0,137	0,137													
12	*Glomeris oblongo 2*	0,152	0,148	0,146	0,146	0,146	0,110	0,110	0,114	0,137	0,137	**0,000**												
13	*Glomeris oblongo 3*	0,152	0,148	0,146	0,146	0,146	0,110	0,110	0,114	0,137	0,137	**0,000**	**0,000**											
14	*Glomeris primordialis 1*	0,151	0,125	0,125	0,125	0,125	0,088	0,088	0,102	0,149	0,149	0,100	0,100	0,100										
15	*Glomeris primordialis 2*	0,142	0,128	0,128	0,128	0,128	0,090	0,090	0,096	0,145	0,145	0,096	0,096	0,096	**0,014**									
16	*Glomeris klugii 1*	0,135	0,142	0,142	0,142	0,142	0,085	0,085	0,104	0,135	0,135	0,094	0,094	0,094	0,085	0,078								
17	*Glomeris klugii 2*	0,132	0,139	0,139	0,139	0,139	0,088	0,088	0,106	0,137	0,137	0,097	0,097	0,097	0,088	0,081	**0,003**							
18	*Glomeris klugii 3*	0,135	0,142	0,142	0,142	0,142	0,085	0,085	0,104	0,135	0,135	0,094	0,094	0,094	0,085	0,078	**0,000**	**0,003**						
19	*Glomeris klugii 4*	0,135	0,142	0,142	0,142	0,142	0,085	0,085	0,104	0,135	0,135	0,094	0,094	0,094	0,085	0,078	**0,000**	**0,003**	**0,000**					
20	*Glomeris klugii 5*	0,140	0,149	0,149	0,149	0,149	0,088	0,088	0,107	0,145	0,145	0,097	0,097	0,097	0,085	0,078	**0,012**	**0,015**	**0,012**	**0,012**				
21	*Glomeris klugii 6*	0,140	0,149	0,149	0,149	0,149	0,088	0,088	0,107	0,145	0,145	0,097	0,097	0,097	0,085	0,078	**0,012**	**0,015**	**0,012**	**0,012**	**0,000**			
22	Glomeris aurita 1*	0,157	0,139	0,143	0,143	0,143	0,104	0,104	0,107	0,131	0,131	0,082	0,082	0,082	0,102	0,099	0,084	0,087	0,084	0,084	0,090	0,090		
23	Glomeris aurita 2*	0,160	0,145	0,151	0,151	0,151	0,107	0,107	0,101	0,137	0,137	0,085	0,085	0,085	0,111	0,107	0,091	0,094	0,091	0,091	0,094	0,094	**0,024**	
24	Glomeris aurita 3*	0,160	0,145	0,151	0,151	0,151	0,107	0,107	0,101	0,137	0,137	0,085	0,085	0,085	0,111	0,107	0,091	0,094	0,091	0,091	0,094	0,094	**0,024**	**0,000**

## Results

### Sequence data and distance analysis

**Figure 1:**
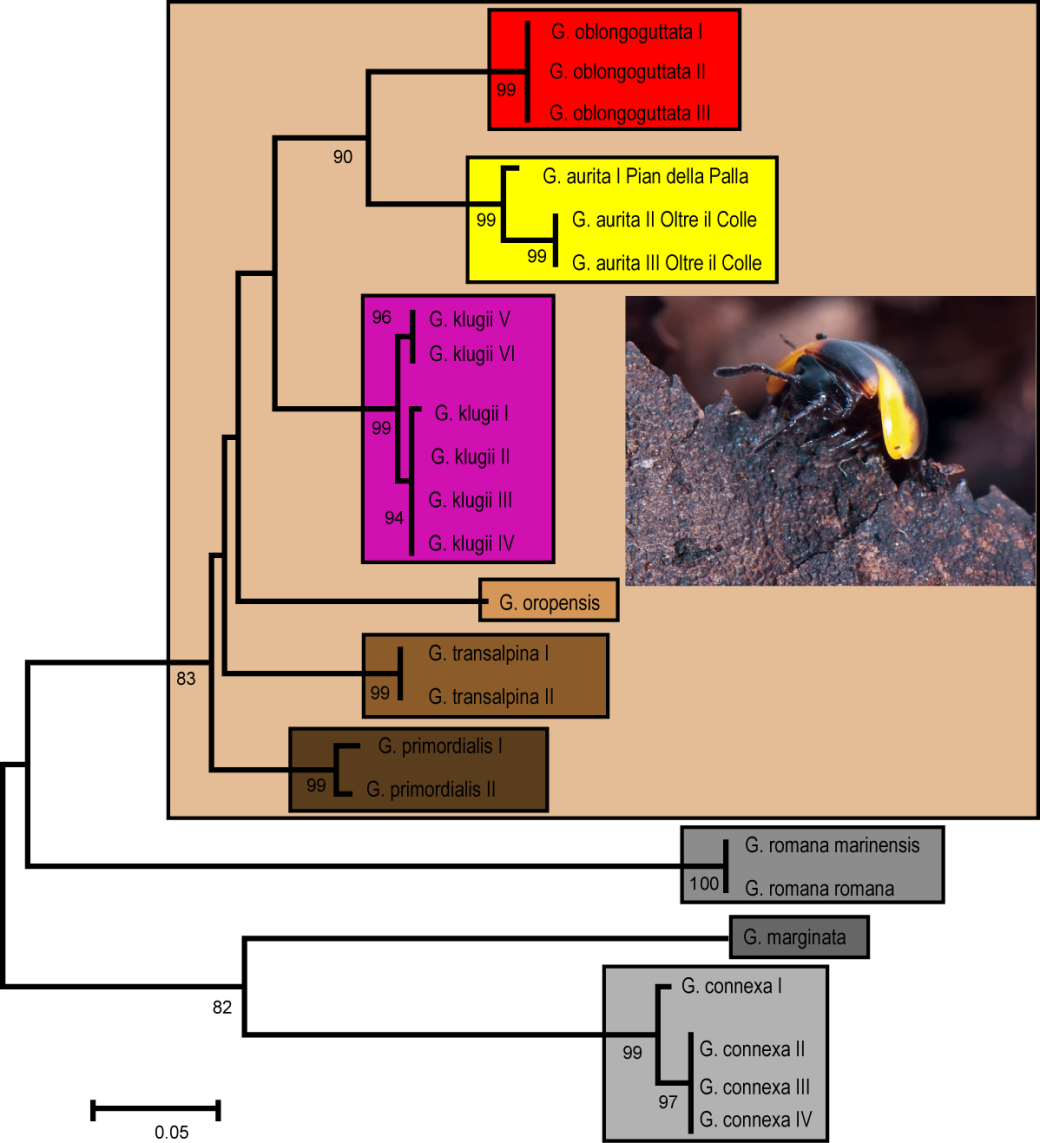
ML Tree calculated after the Hasegawa-Kishino-Yano model with gamma distribution. Each box denominates a species. Golden box = *G. aurita* Koch, 1847. Numbers refer to bootstrap values. Scale bar = 0.05 substitutions/site. Photo taken by J.P. Oeyen, CC-BY 4.0.

Our genetic analysis recovers *G. aurita* in a distinct monophyly with high statistical support (Fig. 1). *G. aurita* is in a well-supported sister-group (86%) with the other Bergamasque endemic *G. oblongoguttata*. Both are standing in a grouping supporting the *G. klugii* species-group, with *G. klugii* followed by *G. oropensis* and *G. transalpina; G. primordialis* is in the basal-most position. The other three species are separate, with *G. romana* in an unsupported sister-group with *G. marginata* and *G. connexa* (Fig. 1).

*G. aurita* shows the lowest interspecific genetic distance to *G. oblongoguttata* (Table 2, 8.2–8.5%) and *G. klugii* (8.4–9.4%), both of which occur in direct syntopy. The two sampled populations of *G. aurita* show an intraspecific variation of 2.4%, with both specimens from the Valle d’Arera showing an identical haplotype.

#### *Glomeris aurita* Koch, 1847

Koch, 1847: 92 (first description); Koch, 1863: 3, pl. 1 (illustration); Berlese, 1883: 7, pl. 7 (list); Brölemann, 1895: 22 (locality); Verhoeff, 1911: 102 (key); Attems, 1927: 252 (key); Manfredi, 1932: 16 (list); Verhoeff, 1936: 226 (list); Wolf, 1938: 483 (list); Attems, 1949: 122 (list); Strasser & Minelli, 1984: (list); Minelli, 1985: 2 (list); Foddai et al., 1995: 11 (list); Kime & Enghoff, 2011: 25 (list)

**Material examined:**

ZFMK: **11 M&F**, **ZFMK MYR110**, Italy: Prov. Bergamo, Oltre il Colle, Piano Palla, locality Forca Larga, N of Skilift, small stand of beeches on steep stony slope, under stones and wood, 1,479 m, 45.87750°N, 9.77765°E, A. Schönhofer leg. 19.09.2009; **1 M, 1 F, ZFMK MYR248**, Italy: Prov. Bergamo, Roncobello, around Corno Branchino and Lago Branchino, alpine meadow, under stones, P. Pantini, A. Schönhofer leg. 17.09.2009, 1,794 m, 45.94931°N, 9.80257°E; **3 F, ZFMK MYR115**, Italy: Prov. Bergamo, Valle Valzurio, Oltressenda Alta, Rifugio Rino Olme, under stones in alpine meadow, 1,814 m, 45.94810°N, 10.03777°E, A. Schönhofer leg. 18.09.2009; **1 M, ZFMK MYR2864**, Bergamo, Oltre il Colle, Valle d’Arera, 2,000 m, 45.9304°N, 9.8046°E, leg. 03.viii.2014, M. Massaro & M. Valle; **3 F, ZFMK MYR2865**, same data as previous; **1 M, ZFMK MYR2866**, same data as previous; **1 F, ZFMK MYR2867**, same data as previous; **5 M&F, ZFMK MYR4097**, same data as previous; **1 F, ZFMK MYR2641**, Bergamo, Oltre il Colle, Piano Palla, locality Forca Larga, N of Skilift, small stand of beeches on steep stony slope, under stones and wood, 1,479 m, 45.87750°N, 9.77765°E, leg. 24.06.2014.

Bergamo Museum: **>15 M&F**, Bergamo, Parre, Pendici meridionali Monte Secco, 2,100 m, 45.927°N, 9.880°E, leg. 23.07.2003; **6 M&F**, Bergamo, Premolo, doline Sud di Baita Camplano, 1,850 m, 45.92°N, 9.83°E, leg. 22.07.2003; **1 ?**, Bergamo, Premolo, macereto, 1,850 m, 45.92°N, 9.83°E, leg. 04.08.2004; >**15 M&F**, Bergamo, Premolo, Forcella di Valmora, 2,000 m, 45.933°N, 9.833°E, leg. 03.07.1983; **2 ?**, Bergamo, Oltressenda Alta, Sotto cresta Valzurio, 1,700 m, 45.956°N, 10.040°E, leg. 09.08.1982; **>15 M&F**, Bergamo, Gromo, Passo di Valle Scura, 2,103 m, 45.976°N, 10.015°E, leg. 19.08.1982; **10 M&F**, Bergamo, Gromo, Baita Alta fontana Mora, 1,950 m, 45.975°N, 10.000°E, leg. 17.06.1984; **2 M, 1 F**; Bergamo, Gandelino, Baita Alta fontana Mora, 2,100 m, leg. 25.7.1984, Bonacina, Lazzaroni, Valle; **9 M&F**, Bergamo, Oltre il Colle, Valle d’Arera, 2,000 m, 45.9304°N, 9.8046° E, leg. 03.viii.2014, Valle; **>15 M&F**, Bergamo, Oltre il Colle, Valle d’Arera, 2,050 m, leg. 22.7.-1.10.2003, pitfalls; **5 M&F**, Bergamo, Oltre il Colle, Valle d’Arera, 2,050 m, leg. 04.08.-29.09.2004, pitfalls; **1 ?**, Bergamo, San Giovanni Bianco, Pozzo sul Monte Cancerno, leg. 02.06.1955, Bonino; **2 M**, Bergamo, Schilpario, Pendici Cimone della Bagoz, 1,700 m, leg. 06.08.-18.10.2005, pitfalls; **1 ?**, Bergamo, San Giovanni Bianco, Pozzo sul Monte Cancerno, leg. 02.06.1955.

**Localities with photographic evidence**: Monte Baldo, 1,900 m, 45.7192°N, 10.8474°E; Monte Frerone, 2,400 m, 45.9418°N, 10.4131°E; Concarena Massif, ∼2,000 m, 46.01°N, 10.25°E; Monte Presolana, southern slope, >1,700 m, 45.949°N, 10.075°E; Passo di San Simone, 2,000 m, 46.0463°N, 9.6853°E.

**Localities from the literature**: Bergamo, Val Brembana, Foppolo (Brölemann, 1895), 46.04°N, 9.76°E, ∼1,600 m.

**Remarks**: Koch’s single known type specimen could not be located, it is not in the ZMB, NHML, ZSM, or UZH collections (personal communications), the usual depositories of type specimens by Koch (Sierwald & Reft, 2004).

**Figure 2:**
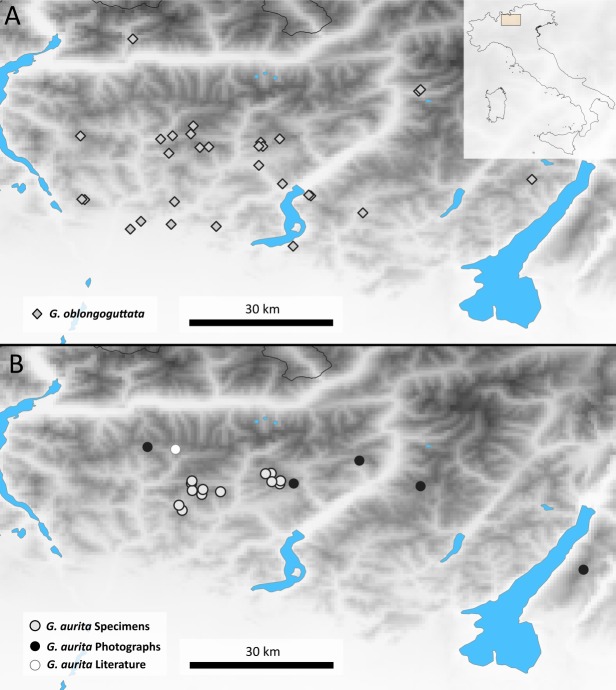
Maps showing the Bergamasque Alps area between the Lago di Como and the Lago di Garda. (A) new localities for *G. oblongoguttata* Verhoeff, 1894; (B) Localities for *G. aurita* Koch, 1847.

**Figure 3:**
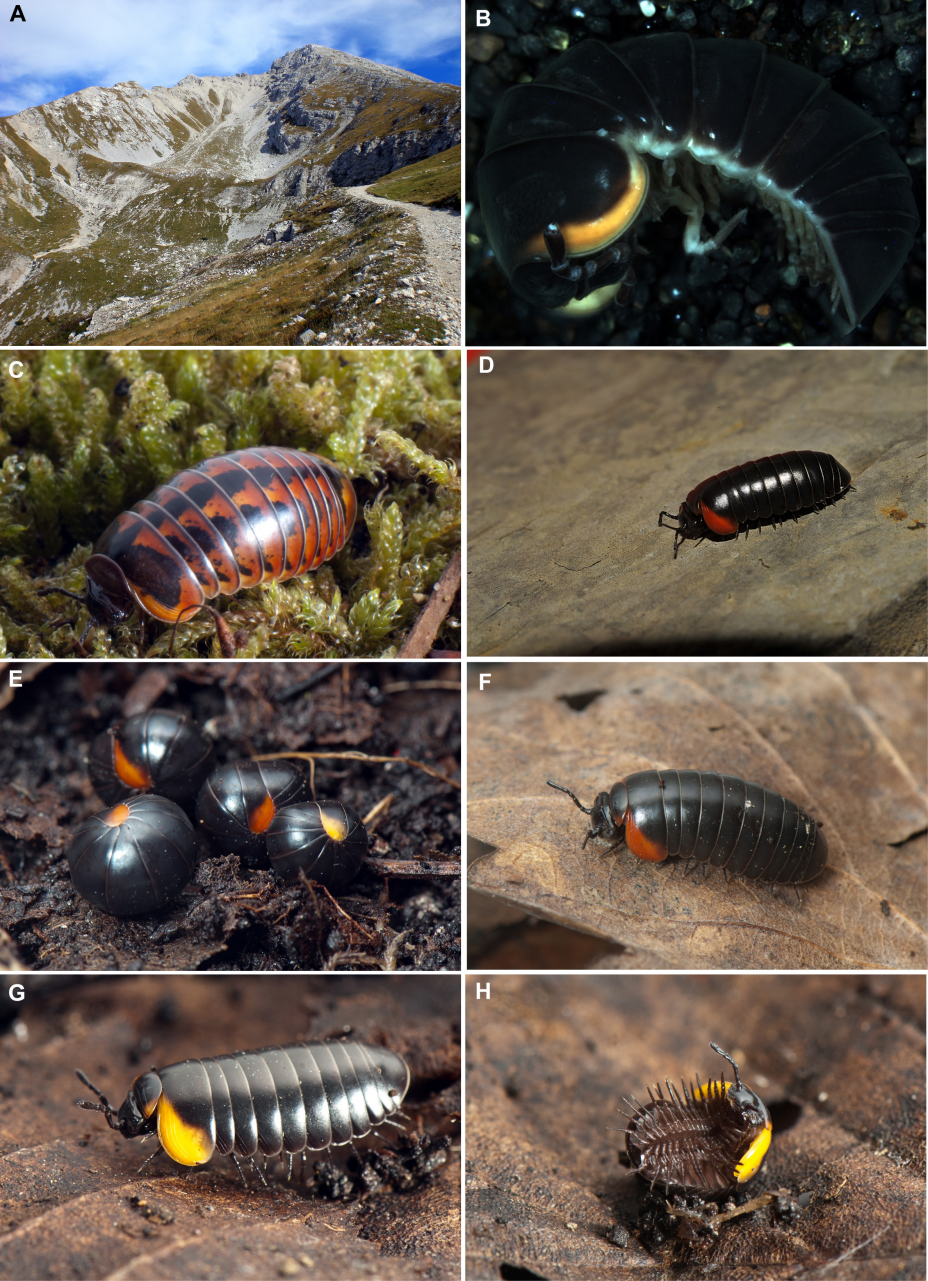
Photographs of the analyzed Glomeris species. (A) Habitat of *G. aurita*, locality close to Monte Arera, photo by M Massaro; (B) Black colour morph of *G. romana*; (C) *G. oblongoguttata*, specimen from Pisogne, photo by JP Oeyen; (D) *G. aurita*, Monte Arera, photo by F Scarlassara; (E) Several specimens of *G. aurita*, rolled-up, showing the yellow and orange colour morph, both from the Monte Arera; (F) *G. aurita*, orange colour morph, walking, specimen from Monte Arera; (G) *G. aurita*, yellow colour morph, walking, specimens from Monte Arera; (H) *G. aurita*, same specimen as in G, ventral side. Photos (E–H) by J.P. Oeyen. All photos CC-BY 4.0.

**Distribution**: Bergamasque Alps from east of Lago di Como to slightly east of the Lago di Garda (Fig. 2), where *G. aurita* is found in exposed mountain meadows (Fig. 3A).

**Differential diagnosis**:

Colour pattern: *G. aurita* resembles in its coloration the black colour morph of *G. romana* (Fig. 3B). Such orange (but mostly yellowish) bands also occur in *G. klugii*, *G. oblongoguttata* (Fig. 3C) and *G. primordialis* as well as in the two high-altitude species *G. transalpina* and *G. oropensis*. However, only in *G. aurita*, the orange (sometimes yellow) band of the thoracic shield expands centrally (Figs. 3D–3H). The whole ventral side of *G. aurita* is black, in most other *Glomeris* species it is more grayish in colour.

Striation: *G. aurita* can be easily distinguished from *G. klugii* and *G. oblongoguttata*, both of which live in syntopy (all other species are not reported from the distribution area of *G. aurita*), in the thoracic shield stria (see Supplementary information 1 for a comparison of *G. aurita* with the syntopic *G. oblongoguttata*). All analyzed and reported specimens of *G. klugii* and *G. oblongoguttata* lack a main stria (one that crosses the thoracic shield), while all analyzed specimens of *G. aurita* have such a main stria.

**Figure 4:**
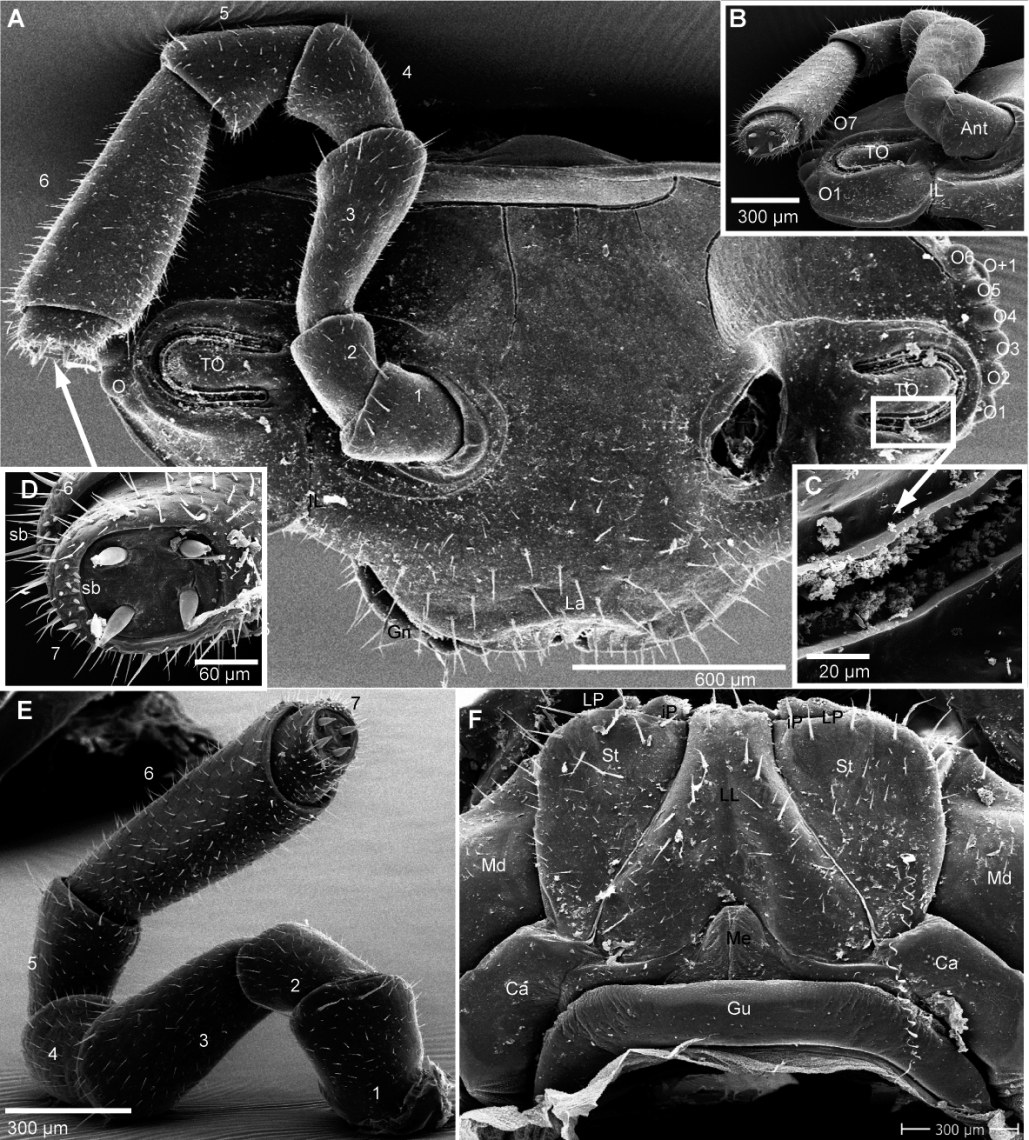
*G. aurita* Koch, 1847, ZFMK MYR248, SEM, male. (A) Head, frontal view; (B) right corner of head, lateral view; (C) detail of organ of Tömösváry; (D) antennomere 7 and disc of right antenna; (E): left antennae, lateral view; (F) gnathochilarium ventral view, *in situ*. Abbreviations: Ant, antenna; Ca, cardines of gnathochilarium; Gn, gnathochilarium; Gu, gula; IL, incisura lateralis; iP, inner palpus; La, labrum; LL, lamellae linguales; LP, lateral palpus; Md, basal joint of mandible; Me, mentum; O, ocelli; sb, sensilla basiconica; St, stipites; str, stria; TO, organ of Tömösváry; numbers refer to antennomeres. Images from the author.

**Figure 5:**
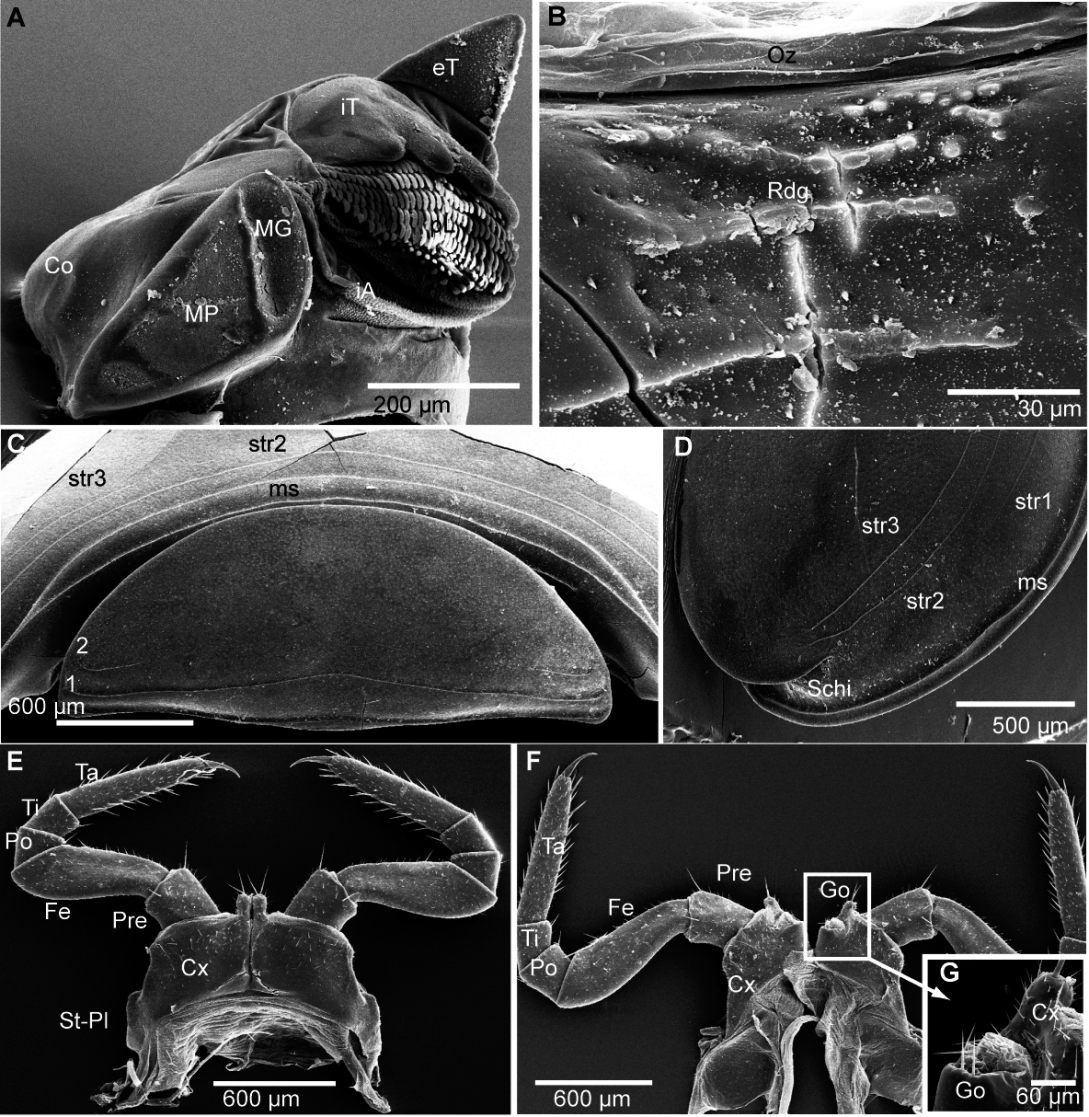
*G. aurita* Koch, 1847, ZFMK MYR248, SEM, male. (A) Left mandible, mesal view; (B) ozopore of tergite 6, dorsal view; (C) collum (tergite 1) and anterior part of thoracic shield (tergite 2), frontal view; (D) thoracic shield, antero-lateral view on schisma; (E) male left leg 1 with fused stigmatic plate, posterior view; (F) male leg 2, posterior view; (G) male gonopores, posterior view. Abbreviations: Co, condylus of mandible; Cx, coxa; eT, external tooth of mandible; Fe, femur: Go, gonopore; iA, intermediate area of mandible; iT, 4-combed inner tooth; MG, molar groove; MP, molar plate; ms, marginal stria; Oz, ozopore; Po, postfemur; Pre, prefemur; Rdg, ridges of ozopore; Schi, schisma; St-Pl, stigmatic plate fused to coxa 1; str, stria; Ta, tarsus; Ti, tibia; numbers refer to number stria. Images from the author.

**Figure 6:**
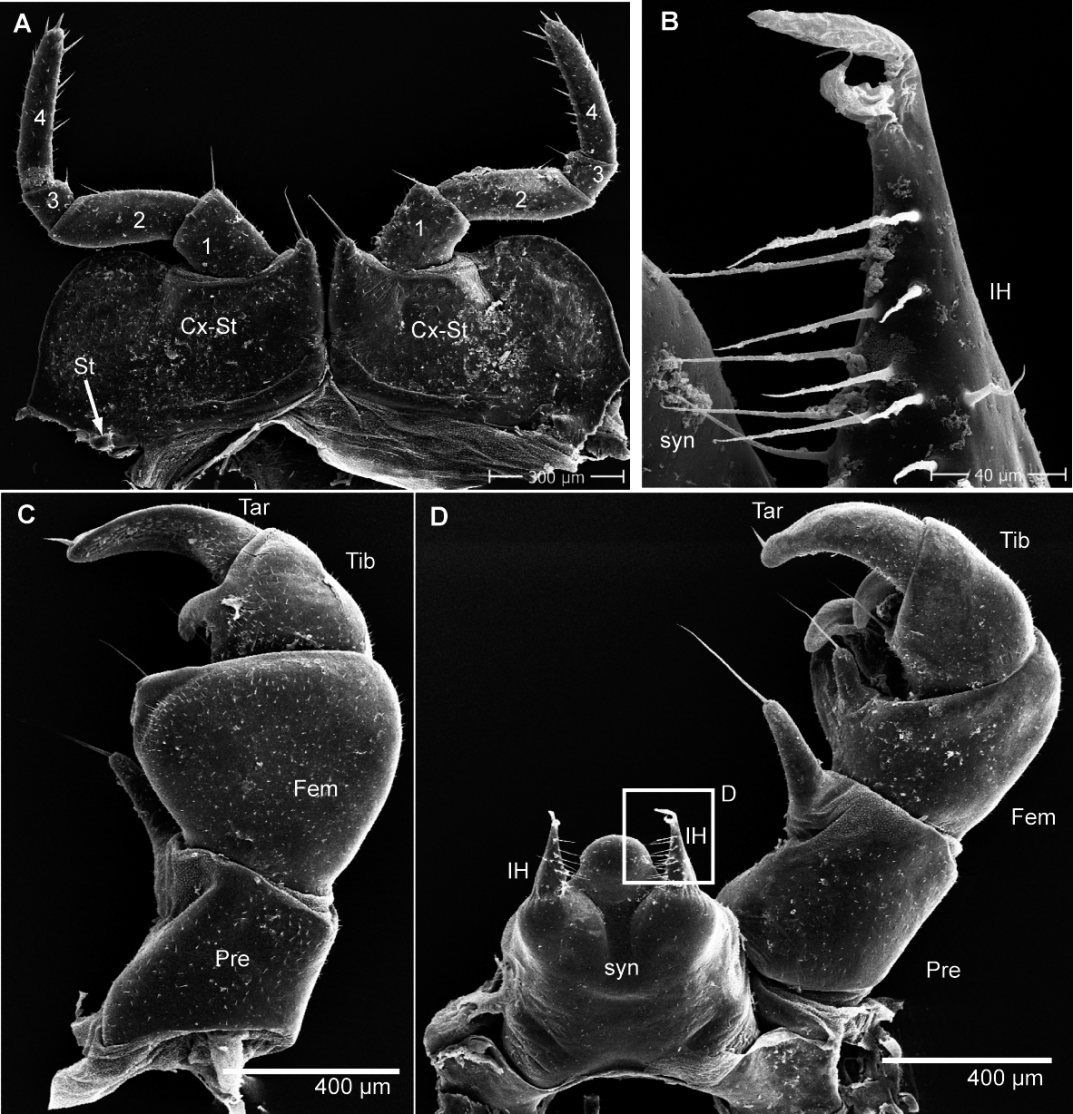
*G. aurita* Koch, 1847, ZFMK MYR248, SEM, male. (A) Male leg pair 17 with fused stigmatic plates, anterior view; (B) right inner horn of posterior telopod; (C) right posterior telopod, posterior view; (D) left posterior telopod with syncoxite, anterior view. Abbreviations: Cx-St, fused coxae 17 with stigmatic plate; Fem, femur; IH, inner horn; Pre, prefemur; St, stigma opening; syn, syncoxite; Ta, tarsus; Ti, tibia. Own illustrations, CC-BY 4.0.

Head (Fig. 4A), organ of Tömösváry (Fig. 4A, 4C), antennae (Figs. 4A–4E), gnathochilarium (Fig. 4F), mandible (Fig. 5A), ozopore (Fig. 5B), collum (Fig. 5C), thoracic shield (Fig. 5D), male leg pair 1 (Fig. 5E), leg pair 2 with gonopore (Figs. 5F, 5G), male leg pair 17 (Fig. 6A), and the telopod (Figs. 6B–6D) with inner horns (Fig. 6B) are illustrated.

**Ecology**: *G. aurita* is unique among known species of *Glomeris* as it was apparently never collected below 1,400 m, but occurs spanning a zone ranging from 1,400–2,400 m, being most commonly recorded at an average elevation of 1,892 m. The habitats are very open mountain meadows (Fig. 3A). While *G. aurita* was commonly found together with widespread species such as *G. connexa* and *G. klugii* and the local endemic *G. oblongoguttata*, all of the three latter species (but not *G. aurita*) could also be found at lower elevations. Kime & Enghoff (2011) mention records, without giving any details, of *G. aurita* from caves. Caves, with their constant cool climate, often show a rich Diplopoda fauna with numerous morphological adaptations (Liu et al., 2017). The studied specimens of *G. aurita*, however, show no sign of cave adaptations.

The activity period of *G. aurita*, at least on the surface, seems to span June to the middle of September, just 3.5 of 12 months of the year.

### *Glomeris oblongoguttata* Verhoeff, 1894

Recent taxonomic review: Hoess & Scholl, 1999; Wesener & Conrad, 2016 (genetic analysis).

**Comments**: As relatively few localities for this Bergamasque endemic (Kime & Enghoff, 2011) are known, additional localities from the collections of the ZFMK and Bergamo Museum are listed below:

**Material examined**:

Bergamo Museum: **1 ?**, Bergamo, Premolo, canalone presso sorgentina Parina, 1,800 m, 45.925°N, 9.825°E, leg. 18.07.1984; **#3**, **1 ?**, Bergamo, Maresana, leg. 03.08.1965; **1 ?**, Bergamo, Astino, leg. 15.11.1964; **#93**, **1 ?**, Bergamo, Bossico, leg. 03.09.1959; **#46**, **1 ?**, Bergamo, Carona, Laghi Gemelli, leg. 15.09.1980; **#29**, **2 ?**, Bergamo, Dossena, Baite del Menna, 1,300–1,400 m, leg. 29.07.1963; **#24**, **2 ?**, Bergamo, Entratico, I Moi, 520 m, leg. 15.-20.06.1957; **#48**, **1 ?**, Bergamo, Oltressenda Alta, Pagherola Bassa, 1,550 m, leg. 09.08.1982; **1 ?**, Bergamo, Premolo, Prateria alpina, 1,900 m, leg. 04.08.-29.09.2004; **3 ?**, Bergamo, Premolo, Baita Camplano, 1,850 m, leg. 22.07.-01.10.2003; **8 M&F**, Bergamo, Premolo, Baita Camplano, 1,850 m, leg. 19.06.-22.07.2003; **#75**, **1 ?**, Bergamo, Roncobello, leg. 29.06.1970; **#80**, **5 M&F**, Bergamo, Roncobello, torrente Valsecca, leg. 01.06.1956; **#61**, **2 ?**, Bergamo, Selvino, 1,000 m, leg. 10.08.1959; **#54**, **3 ?**, Bergamo, Torre de Busi, Valcava-Monte Albenza, leg. 26.09.1956; **#8**, **8 M&F**, Bergamo, Torre de Busi, Valcava, 1,300 m, leg. 19.08.1959; **#2**, **6 M&F**, Bergamo, Villa di Serio, Colli, leg. 23.09.1958; **5 M&F**, Sondrio, Val Masino, Filorera, in ghiaione, 1,000 m, leg. 01.07.1984; **#60**, **3 ?**, Brescia, Ponte Saviore, in pineta sopra il mulino, leg. 23.08.1963; **#44**, **1 ?**, Brescia, Ponte Saviore, in pineta sotto sassi, leg. 21.07.1963; **#49**, **4 ?**, Brescia, Ponte Saviore, leg. 31.07.1964.

ZFMK: **ZFMK-MYR2486**, **8 M&F**, Brescia, Pisogne, Bachtal im ehemaligen Minenbetrieb, Fußweg zum Wasserfall, südlich Bach, kärglich bewachsener steiler NE Hang mit einzelnem Haselnußstrauch, 403 m, 45°47′46.14″N, 10°7′14.68″E, leg. 07.10.2012; **ZFMK-MYR253**, **3 F**, Trient, Passo di Tremalzo, mainly under stones and wood in forest, 900-1500 m, 45.84N, 10.71E, leg. 11.08.2009; **ZFMK-MYR2647**, **1 ?**, Bergamo, Valle Valzurio, Oltressenda Alta, oberhalb Spinellis, Wanderweg zum Refugio Rino Olme (Bergamo IIIA), nördlicher Hang nahe Gebirgsbach, unter Laub auf Felsbrocken, 1,000 m, 45° 55′41.20″N, 9°59′34.78″E, leg. 26.06.2014; **ZFMK-MYR2648**, **1 ?**, same data as previous; **ZFMK-MYR619**, **8 M&F**, Brescia, Lago di Iseo, Pisogne, Quarry with lots of soft calcareous stones, sparse Populus only vegetation, under stones, 281 m, 45.79851°N, 10.11522°E, leg. 09.04.2011; **ZFMK-MYR159**, **1 F**, Bergamo, Valle Valzurio, Oltressenda Alta, Spinelli, rich structured low growing deciduous forest, under stones and sieved from leaf and wood litter, 933 m, 45.92856N, 9.98221E, leg. 18.09.2009; **ZFMK-MYR2639**, **1 ?**, Bergamo, Oltre il Colle, Piano Palla, locality Forca Larga, N of Skilift (Bergamo I), small stand of beeches on steep stony slope, under stones and wood, 1,479 m, 45.87750N, 9.77765E, leg. 24.06.2014; **ZFMK-MYR2640**, **1 ?**, same data as previous; **ZFMK-MYR2667**, **1 ?**, same data as previous; **ZFMK-MYR2668**, **1 ?**, same data as previous; **ZFMK-MYR2669**, **1 ?**, same data as previous; **ZFMK-MYR2670**, **1 ?**, same data as previous; **ZFMK-MYR2671**, **1 ?**, same data as previous; **ZFMK-MYR2672**, **1 ?**, same data as previous; **ZFMK-MYR2673**, **1 ?**, same data as previous; **ZFMK-MYR2674**, **1 ?**, same data as previous; **ZFMK-MYR2675**, **1 ?**, same data as previous; **ZFMK-MYR2676**, **1 ?**, same data as previous; **ZFMK-MYR2677**, **1 ?**, same data as previous; **ZFMK-MYR2678**, **1 ?**, same data as previous; **ZFMK-MYR2679**, **1 ?**, same data as previous; **ZFMK-MYR2680**, **1 ?**, same data as previous; **ZFMK-MYR2681**, **1 ?**, same data as previous; **ZFMK-MYR2682**, **1 ?**, same data as previous; **ZFMK-MYR2683**, **1 ?**, same data as previous; **ZFMK-MYR2684**, **1 ?**, same data as previous; **ZFMK-MYR2685**, **1 ?**, same data as previous; **ZFMK-MYR2686**, **1 ?**, same data as previous; **ZFMK-MYR2687**, **1 ?**, same data as previous; **ZFMK-MYR2688**, **1 ?**, same data as previous; **ZFMK-MYR2689**, **1 ?**, same data as previous; **ZFMK-MYR2690**, **1 ?**, same data as previous; **ZFMK-MYR2691**, **1 ?**, same data as previous; **ZFMK-MYR2692**, **1 ?**, same data as previous; **ZFMK-MYR2693**, **1 ?**, same data as previous; **ZFMK-MYR2696**, **1 ?**, same data as previous; **ZFMK-MYR** **2697**, **1 ?**, same data as previous; **ZFMK-MYR2698**, **1 ?**, same data as previous; **ZFMK-MYR2699**, **1 ?**, same data as previous; **ZFMK-MYR2700**, **1 ?**, same data as previous; **ZFMK-MYR2727**, **1 ?**, same data as previous; **ZFMK-MYR2728**, **1 ?**, same data as previous; **ZFMK-MYR2729**, **1 ?**, same data as previous; **ZFMK-MYR2730**, **1 ?**, same data as previous; **ZFMK-MYR2642**, **1 ?**, Bergamo, NE Roncobello (Bergamo II), steinige Alpenvegetation nahe Schneefeld, 1,600 m, 45.961023N, 9.800735E, leg. 25.06.2014; **ZFMK-MYR2643**, **1 ?**, same data as previous; **ZFMK-MYR2644**, **1 ?**, same data as previous; **ZFMK-MYR2645**, **1 ?**, same data as previous; **ZFMK-MYR2646**, **1 F**, same data as previous; **ZFMK-MYR2733**, **1 ?**, same data as previous; **ZFMK-MYR2734**, **1 ?**, same data as previous; **ZFMK-MYR2735**, **1 ?**, same data as previous; **ZFMK-MYR2736**, **1 ?**, same data as previous; **ZFMK-MYR2737**, **1 ?**, same data as previous; **ZFMK-MYR2738**, **1 ?**, same data as previous; **ZFMK-MYR2739**, **1 ?**, same data as previous; **ZFMK-MYR2740**, **1 ?**, same data as previous; **ZFMK-MYR2741**, **1 ?**, same data as previous; **ZFMK-MYR2742**, **1 ?**, same data as previous; **ZFMK-MYR2743**, **1 ?**, same data as previous; **ZFMK-MYR2744**, **1 ?**, same data as previous; **ZFMK-MYR2745**, **1 ?**, same data as previous; **ZFMK-MYR2746**, **1 ?**, same data as previous; **ZFMK-MYR2747**, **1 ?**, same data as previous; **ZFMK-MYR2748**, **1 ?**, same data as previous; **ZFMK-MYR2749**, **1 ?**, same data as previous; **ZFMK-MYR2750**, **1 ?**, same data as previous; **ZFMK-MYR2751**, **1 ?**, same data as previous; **ZFMK-MYR2752**, **1 ?**, same data as previous; **ZFMK-MYR2753**, **1 ?**, same data as previous; **ZFMK-MYR2754**, **1 ?**, same data as previous; **ZFMK-MYR2755**, **1 ?**, same data as previous; **ZFMK-MYR2756**, **1 ?**, same data as previous; **ZFMK-MYR2757**, **1 ?**, same data as previous; **ZFMK-MYR2758**, **1 ?**, same data as previous; **ZFMK-MYR1692**, **1 ?**, Brescia, SP50 Tavernole Sul Mella - Ville (Marmentino), Localita Molino, trockener Hang mit Brombeeren. unter Grasbüschel, 660 m, 45° 45′2.99″N, 10°15′34.32″E, leg. 24.04.2013; **ZFMK-MYR213**, **6 M&F**, Bergamo, Valle Valzurio, Oltressenda Alta, Rifugio Rino Olme, under stones in alpine meadow, 1,814 m, 45.94810N, 10.03777E, leg. 18.09.2009; **ZFMK-MYR2649**, **1 ?**, Bergamo, Valle Valzurio, Oltressenda Alta, Refugio Rino Olme (Bergamo IIIB), under stones in alpine meadow, 1,814 m, 45.94810N, 10.03777E, leg. 26.06.2014; **ZFMK-MYR232**, **>8 M&F**, Bergamo / Como, Zucco Barbesino, S side of Valle de Camosci, under stones in alpine meadows and stony gravel, 1,815 m, 45.95576N, 9.50679E, leg. 24.09.2009; **ZFMK-MYR1693**, **1 ?**, Brescia, Lago di Iseo, SP48 Iseo - Polaveno, oberhalb Bosine, Westhang mit Eschen und Brombeeren, eher Trockenvegetation. unter Moos 440 m, 45°39′43.49″N, 10° 4′25.19″E, leg. 24.09.2013.

## Discussion

### Comparison of the micro-CT scan with scanning electron microscopy

**Figure 7:**
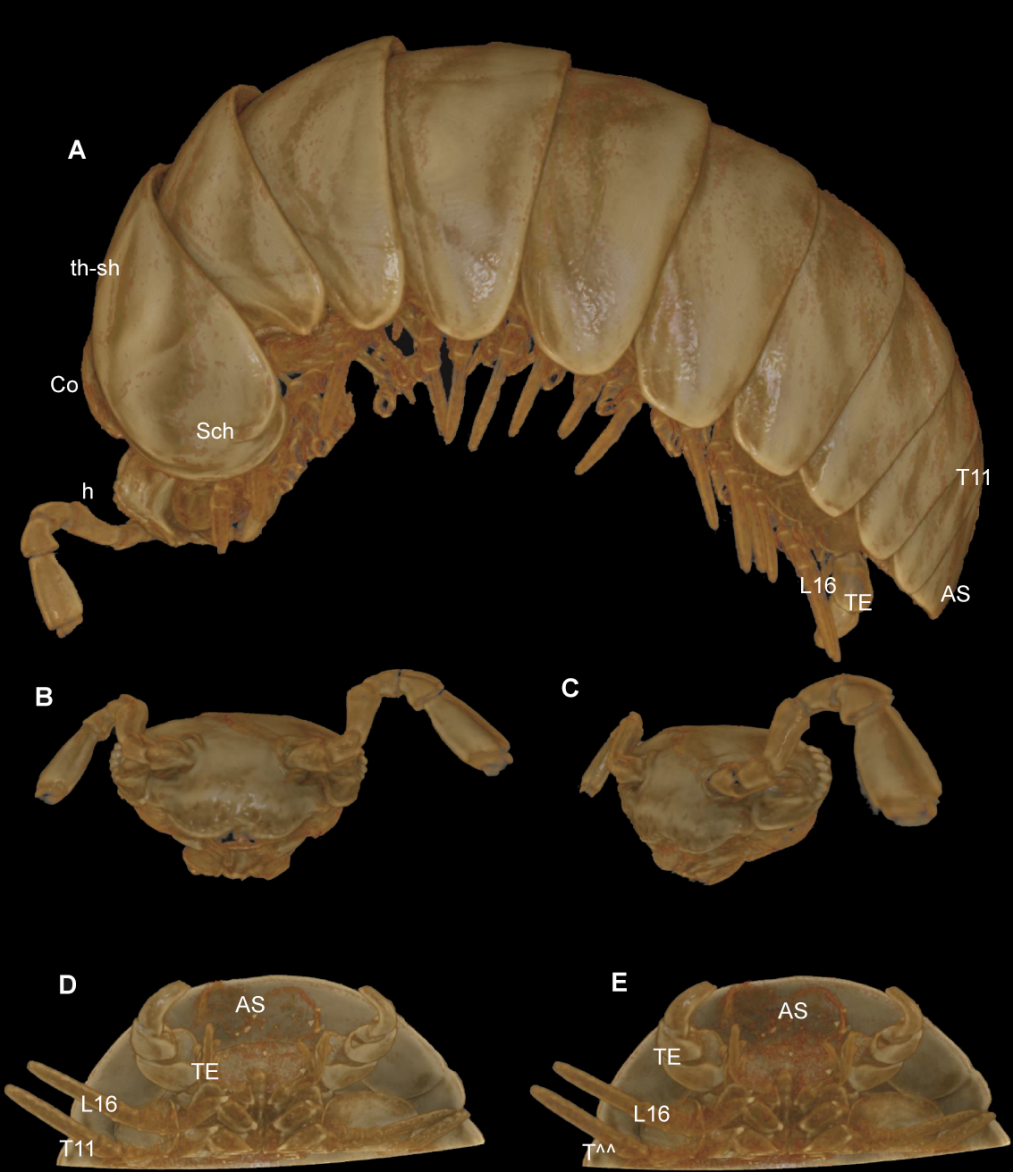
*G. aurita* Koch, 1847, ZFMK MYR248, CT scan, male. (A) Habitus, lateral view; (B) digitally isolated head, frontal view; (C) digitally isolated head, lateral view; (D) posterior body end, ventral view, strong filter; (E) posterior body end, ventral view, weak filter. Abbreviations: AS, anal shield; Co, collum (tergite 1); h, head; L16, leg 16; Sch, schisma of thoracic shield; T11, tergite 11; TE, telopods; th-sh, thoracic shield (tergite 2). Not to scale. Illustrations prepared by Leif Moritz (Bonn), CC-BY 4.0.

The micro-CT (Fig. 7), conducted with a specimen still in the ethanol-filled collection vial, was obviously less invasive than the SEM scans, for which a specimen had to be dissected, dried, and sputtered with gold. The CT-scan, including the formatting, and the rendering (without any manual reconstruction), was as time consuming as the SEM, taking approximately 8 working hours. The SEM sample took 2 hours of preparation/dissecting, 2 hours for mounting, and 3 hours for taking the micrographs.

The results are difficult to compare. The SEM images show a much greater detail, including spines and setae (see Figs. 4–6), which are of taxonomic importance. In the SEM images, the striae on the thoracic shield (Fig. 5D) and the transverse striae of the collum (Fig. 5C) are clearly visible, which is not the case in the CT images (Figs. 7A–7E).

Critical point drying of the specimen would probably have yielded better scan results, as was done in previous taxonomic studies (Akkari, Enghoff & Metscher, 2015; Liu, Rühr & Wesener, 2017; Sagorny & Wesener, 2017; Moritz & Wesener, 2017), but this would impact the whole specimen and no longer be a minimally invasive technique. Manual reconstruction of certain body parts could have resulted in better illustrations, e.g., of the telopods. However, such reconstructions are time-consuming. For taxonomic descriptions of millipedes, while certainly adding important information and a digital specimen to the descriptions, it does not seem possible for the CT technology to replace more traditional techniques such as SEM.

### Evolutionary history of *G. aurita*, a high-altitude endemic

Interestingly, all central European high-altitude pill millipede species show a local, restricted distribution in different areas of the Southern Alps. None occurs in the central or northern Alps (Kime & Enghoff, 2011). This striking pattern might be connected with glaciations of the Alps, which only spared the southern-most mountains (Penck & Brückner, 1909; Holdhaus, 1954). At least for soil arthropods, such as pill millipedes of the genus *Glomeris*, these southern, ice-free mountains formed viable refugia allowing the survival of local endemics in climatically unsuitable times.

### *G. aurita*—a Nunatak pill millipede?

The closely related species *G. oblongoguttata*, a regional endemic of almost the same areas (Hoess, 2000) as *G. aurita*, occurs in some places syntopic to *G. aurita*, but is also found at much lower elevation. The fact that *G. aurita* is rarely found below 1,500 m, and at high-altitude often close to snow fields and some of the coldest microclimatic spots (own observation) are hints that *G. aurita*, unlike *G. oblongoguttata*, might represent a Nunatak survivor. The species might not have retreated to warmer refugia during glaciations, but potentially survived above the glaciers on mountain tops. The observed genetic difference between the two sampled *G. aurita* populations (2.4%), despite being separated by less than 5 km (albeit divided by a valley), might be a further indication of such Nunatak endemism. Nunatak survivors are known from the area, such as the beetle *Byrrhus focarilei* (Fabbri & Pütz, 1997) endemic to the Monte Vigna Vaga in the Bergamasque Alps, and the carabid beetles *Boldoriella brembana* (Binaghi, 1937), *Broscosoma relictum* Weissmandl, 1935, and *Dyschirius schatzmayri* Bari, 1950 all from the Monte Arera and Monte Alben area where *G. aurita* occurs. Furthermore, both the Prealpi Bergamasche and the Alpi Guidicarie are known the two centers of plant microendemism (seven and five species, respectively, Tribsch, 2004) in the Alps. Further genetic studies involving more specimens of *G. aurita* from other mountaintops will be necessary to substantiate this hypothesis.

### Biogeographic evaluation—museum collections and citizen scientists

Aside from the single literature record and three samples that came as by-catch in our possession, the majority of locality records (10) of *G. aurita* came from museum collections (Fig. 2). Given the few known localities where *G. aurita* was collected, the five new localities shown by photographic evidence via the Italian Naturalist forum (NaturaMediterraneo, 2018) significantly expand our knowledge about the distribution of the species. While most localities with citizen science evidence fall in between the localities of museum specimens, the Monte Baldo locality is located to the east of the area.

This population of *G. aurita* is of special importance, as it is the only locality east of the Lago di Garda (Fig. 2). During the last Ice Age, Monte Baldo was a Nunatak, completely isolated by the Adige glacier from other areas (Latella, Verdari & Gobbi, 2012), with glaciers reaching as high up as 1,400 m (Penck & Brückner, 1909). Monte Baldo is rich in local endemic invertebrates, including other millipedes such as *Osellasoma caoduroi* (Mauriés, 1984), but also numerous plants and insects such as the grasshopper *Pseudoprumna baldensis* (Krauss, 1883). A morphological and molecular analysis of *G. aurita* specimens of Monte Baldo is greatly encouraged to see whether or not the photographed pill millipedes belong to *G. aurita* or represent a local endemic. Given the known isolation of well-researched Monte Baldo during the last Ice Age, it might be possible to date the observed genetic distances of the Monte Baldo population to those of other populations. This dating might allow a more precise dating of the speciation events of the genus *Glomeris* in northern Italy and beyond to finally gain a better understanding of the pill millipede microendemism so prevalent in the area (Wesener & Conrad, 2016).

## Conclusion

For Italian micro-endemic pill millipedes, the seven species of the *G. dorsosanguine* species-group (*G. dorsosanguine*, *G. judicaria*, *G. longaronensis*, *G. sanguinicolor*, *G. schubarti*, *G. solis*, and *G. strasseri*) remain dubious. All seven species are found in the mountains of SE Italy, most of them only found once or twice. In their general colour pattern, some of those species resemble *G. aurita*. In addition, some other morphological characters used to separate these species by Verhoeff, such as an incomplete second stria on the collum, varies strongly within the here studied *G. aurita* specimens. Our studies (Wesener, 2015b; Wesener & Conrad, 2016; this study) confirm that only new collections of these species and subsequent molecular studies will be able to solve the mysteries surroundings the high number of local endemic pill millipedes in northern Italy.

##  Supplemental Information

10.7717/peerj.5569/supp-1 Supplemental Information 1 Striation of the thoracic shield Striation measured in all specimens of Glomeris aurita and Glomeris oblongoguttata, sorted by locality.

10.7717/peerj.5569/supp-2 Supplemental Information 2 Sequence alignment of the analysis in FASTA format Sequence dataset used for all analysis.

## Abbreviations

NHML: The Museum of Natural History, London, England
UZH: Zoologisches Museum der Universität Zürich, Switzerland
ZFMK: Zoological Research Museum A. Koenig, Bonn, Germany
ZMB: Museum für Naturkunde, Berlin, Germany
ZSM: Zoologische Staatssammlung München, Germany

## Additional Information and Declarations

### DNA Deposition

The following information was supplied regarding the deposition of DNA sequences: The sequences are provided at GenBank under accession numbers MH574905 –MH574907 and also as a Supplemental File.
